# Taken on Trust

**Published:** 1893-01-14

**Authors:** 


### Passing Topics.

### TAKEN ON TRUST.

However conscious we may be of the value of publicity in respect of the affairs of our hospitals, we are occasionally inclined to regret that the law does not afford them some adequate protection against the manufacture or adoption of false accusation. Mr Egmont Hake has righteously deprecated in "Suffering London" the publication of statements calculated to reflect upon the management of our hospitals until at least some proof of their truth is forthcoming, and he very pertinently observes that "a correction of misstatement in one issue of a journal, which has found its way into a previous issue does not always obliterate the impression produced." We might go so far as to say that is never does. A great part of mankind judges hastily, is usually prone to think the worst is likeliest, and attaches to any printed statement a large measure of infallibility.

Two hospitals, Charing Cross and the Poplar Hospital for Accidents, are the last to suffer, and each has substantial occasion for complaint. Both have been referred to prominently upon the contents bills, and in the columns of evening and weekly contemporaries, under the inviting head of "Another Hospital Scandal," whereas in neither instance has there been any scandal at all except such as arises from a want of veracity upon the part of the accusers.

In the one case, the hospital was held up to reprobation for having sent away a patient "to die," whereas the poor man persisted in refusing the proffered admission, although warned of the probable consequences, and in the other, the authorities appear to have done nothing more than remove to the parish infirmary a violent patient whose violence they had no adequate means of controlling, and who, if not already a source of danger to himself and other people, must soon have become so.

No one can question it is a hard thing that institutions which depend for their existence upon the goodwill and confidence of their subscribers should be injured by unwarranted misrepresentations of facts and yet possess no method of obtaining redress. For our own part we think the indignation of the chairman of the Poplar Hospital was amply called forth by the provocation received, and was the more justified because he had been at considerable personal trouble to ensure that the patient in question should be properly provided for. The sarcastic and sapient inquiry of the foreman of the jury at the inquest is worthy of record. "Why," asked this gentleman, "do the public subscribe to hospitals if it is not to keep patients out of the workhouse?" Well, oft-quoted statistics show that the "public" cannot be described with accuracy as hospital-subscribing, while the good Samaritans who do subscribe have scarcely the intention attributed to them. That hospitals keep large numbers of people off the rates, and thus perform a great economic service to the State, is most true, but it is as an indirect result of their beneficent work, which, primarily, is to make sick people well, and thus to save them from becoming a burden upon their fellow-citizens. Ratepayers who have a clear perception of what the hospitals do in this direction ought to find an additional reason for supporting them liberally, and our captious friend should look to it that his subscriptions do not fall short of his magnificent conception of the hospital programme—the extinction of pauperism. Agricola.

